# Intraoperative Tibia Fractures During Primary Total Knee Arthroplasty

**DOI:** 10.7759/cureus.17017

**Published:** 2021-08-09

**Authors:** Aaron Seidman, Adam Green, Daniel McCall, Joseph Finch, Logan C Smith

**Affiliations:** 1 Orthopedic Surgery, Beaumont Hospital Farmington Hills, Farmington Hills, USA

**Keywords:** intraoperative, tibia, fracture, arthroplasty, long-stemmed, component

## Abstract

Intraoperative tibia fractures during primary total knee arthroplasty (TKA) are a known complication, however, the management options and outcome are poorly represented in the orthopedic literature. We performed a thorough literature review and detailed two cases of intraoperative tibia fractures during primary TKA at our institution to determine the mechanism behind these injuries and the optimal treatment strategy. We describe one case of intraoperative tibia fracture during primary posterior stabilized (PS) TKA and one case during primary cruciate retaining TKA. Open reduction and internal fixation with plate and screws, lag screws alone, suture anchors, or tension wiring were the most common treatment options reported in the literature. Although there was no consensus on the optimal postoperative protocol, in our series, both cases achieved satisfactory clinical outcomes with and without further fixation of the fracture. Intraoperative tibia fractures are unforeseeable complications during routine primary TKA and the operating surgeon should be aware of the different strategies in managing these complications.

## Introduction

Total knee arthroplasty, also known as total knee replacement, is the gold standard operative treatment of end-stage osteoarthritis of the knee joint. Over 600,000 procedures were performed across the United States in 2017 and that number continues to rise [1-2]. Total knee arthroplasty (TKA) continues to be one of the most successful surgical procedures in the entire medical field with regard to patient satisfaction and improvement in lifestyle and function [2]. As the number of arthroplasty procedures performed continues to rise, so too will the number of complications, especially given the increase in primary arthroplasty performed in patients younger than 65 [1, 3]. Preventive measures taken preoperatively greatly reduce the risk of major complications such as blood loss, infection, and venous thromboembolism [4-5]. Other complications, such as those encountered intra-operatively, require a quick response and careful attention by the operating surgeon to ensure the optimal outcome [5].

We think that the incidence, treatment, and outcomes of intraoperative tibia fractures during primary TKA are poorly represented in the orthopedic literature. A thorough literature review yields limited publications concerning intraoperative tibia fractures during primary TKA. At our institution, we experienced two intraoperative tibia fractures during primary TKA. We present two cases to aid in proper decision-making when encountering this problem in the operating room, as well as a literature review surrounding intraoperative tibia fractures sustained during primary TKA.

## Case presentation

Case 1

**Figure 1:**
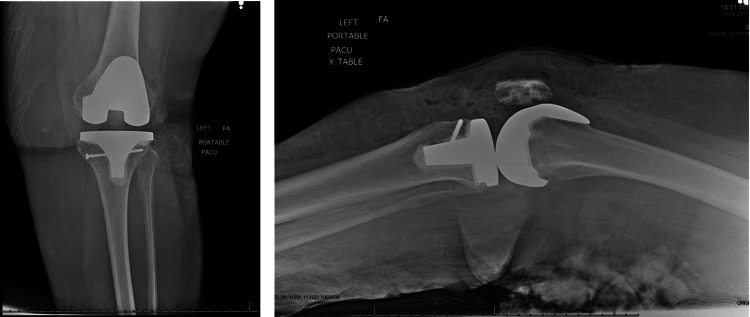
Anteroposterior (Left) and lateral (Right) postoperative radiograph demonstrating a left PS with cancellous screw fixation of an intraoperative tibia fracture. TKA, total knee arthroplasty; PS, posterior stabilized

The first patient is a 72-year-old female with osteoarthritis of the left knee. The patient failed nonoperative treatment modalities including several intra-articular cortisone injections and underwent primary TKA. During the cementation phase, the patient sustained a vertical fracture through the anterior medial plateau. The tibial component remained stable on examination. A standard fracture reduction clamp was used to reduce and stabilize the fracture while we placed a 4.0 mm x 60 mm cancellous screw across the fracture site for stability. Postoperative protocols were unchanged and the patient was allowed and encouraged to weight bear as tolerated and work with physical therapy as planned. The patient showed no functional deficits and no increased pain at her two weeks and two months postoperative visit. Figure 1 shows a postoperative anteroposterior and lateral radiograph of cancellous screw fixation of an intraoperative tibia fracture.

Case 2

**Figure 2:**
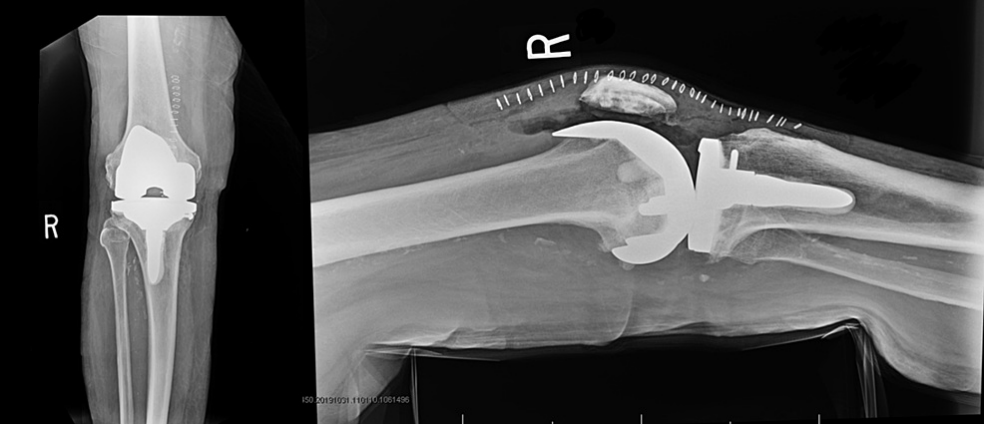
Anteroposterior (Left) and lateral (Right) postoperative radiograph demonstrating a right cruciate retaining TKA with a long stemmed tibial component bypassing the intraoperative tibia fracture. TKA, total knee arthroplasty

The second patient is a 89-year-old male with osteoarthritis of the right knee. The patient failed nonoperative treatment modalities including several intra-articular cortisone injections and underwent primary TKA. During the broaching process, a crack was noted in the sclerotic lateral tibial plateau. The fracture was assessed to be stable. A 4.0 mm x 60 mm cancellous screw with a washer was introduced in the lag technique and good compression was achieved across the fracture site. The fracture was once again assessed after insertion of the screw and was found to be stable. A decision was made to use a long-stemmed tibial component that bypassed the fracture for additional stabilization due to severe osteopenic bone. Routine postoperative weight bearing and physical therapy regimens were initiated. The patient was seen four weeks and two months postoperatively and was without functional deficits or increased pain. Figure 2 shows a postoperative anteroposterior and lateral radiograph of a TKA in which an intraoperative tibia fracture was treated with a stemmed tibial component bypassing the fracture site.

## Discussion

The first report of intraoperative tibia fractures was defined by Stuart and Hanssen and included a classification system based on the anatomic pattern, prosthesis stability, and timing of the fracture [6]. In their review, 19 intraoperative tibia fractures were identified [6]. Type 1 fractures were treated with cancellous screws, Type 2 fractures were treated with stem bypass with bone grafting, Type 3 fractures were treated with plate and screws, and Type 4 fractures were treated with suture fixation [6]. Despite its descriptive and communicative usefulness, this fracture classification system does not correlate with treatment options or predict clinical outcomes. Therefore, a critical review of the available literature was performed to highlight different treatment options and how these decisions affected postoperative outcomes and patient care protocols.

Alden et al. reviewed 17,389 primary TKAs between 1985 and 2005, identifying 66 patients with 67 intraoperative fractures during index arthroplasty procedures, only 18 of which involved the tibia [7]. These results were in accordance with previous reports supporting that intraoperative fracture is more common in females (80%) and less common in the tibia compared to those involving the femur, 73% and 27% respectively [7]. They also noted a majority of these fractures were sustained during exposure and bone preparation and trialing of components, which differed from our patients as all fractures were sustained during the final implant/cementation process.

Agarwala et al. series includes a review of 3,168 primary cemented PS TKAs [8]. Their study included 19 patients with intraoperative fractures, 15 of which involved the tibia [8]. Fractures involving the lateral cortex were reduced, clamped, and fixed with screws or sutures. Those involving the medial cortex with fracture lines extending the planned position of the tibial keel were fixed by placing a stemmed tibial component that extended the fracture site [8]. Postoperative weight-bearing and physical therapy protocols were maintained as if no fractures had incurred, and no difference was found in instability rate, infection, patellar maltracking, loosening, or osteolysis at six months [8].

Pun et al. reviewed 1364 primary cemented PS TKAs performed by a single surgeon, observing 12 intraoperative tibial fractures, all of which were sustained while hammering down the final cemented component [9]. All 12 fractures in these series involved the anterior cortex of the medial plateau and were immediately fixed with 3.5 mm partially threaded cancellous screws [9]. All 12 patients achieved bone union and good function [9].

Finally, Pinaroli et al. reviewed 1,795 PSTKA cases with 40 intraoperative fractures, 30 of which sustained to the tibia [10]. Twenty-seven of these fractures occurred within the tibial plateau, and their choice of fixation included converting to a long keel or stemmed tibial component which passed the fracture site in addition to placing two screws and a cable in a tension band construct for additional stability [10]. In their series, the occurrence of intraoperative fractures did not modify the survival curve of implants at seven-year follow-up [10].

## Conclusions

As the number of primary TKAs performed continues to rise, so too will the intraoperative complications and the incidence of intraoperative tibia fractures. It is important for the performing surgeon to be aware of the incidence, treatment options, and effects on implant survival, and to be able to adequately identify, assess, and address these complications. The classification system proposed by Stuart and Hanssen accurately categorizes the different types of intraoperative tibia fractures, which may aid in future research on this subject as more cases are identified. Treatment decisions, however, are based on the stability of the implant in the setting of intraoperative fracture.

Our collection of case studies demonstrates three different treatment options for intraoperative tibia fractures, all demonstrating good functional outcomes without having to change postoperative weight-bearing status or physical therapy regimens, both of which are vital for the success of the procedure. Cancellous lag screw fixation, employing the use of long-stemmed tibial components to bypass the fracture site, and tension band fixation of fractures, all show to be viable treatment options and are also supported by the existing literature. There is currently no evidence that suggests intraoperative fracture shortens the expected life span of the implant. Further studies are needed to assess the longevity of TKA implants after intraoperative fracture fixation.
